# A Plant-Produced Virus-Like Particle Displaying Envelope Protein Domain III Elicits an Immune Response Against West Nile Virus in Mice

**DOI:** 10.3389/fpls.2021.738619

**Published:** 2021-09-13

**Authors:** Jennifer Stander, Aleyo Chabeda, Edward P. Rybicki, Ann E. Meyers

**Affiliations:** ^1^Biopharming Research Unit, Department of Molecular and Cell Biology, University of Cape Town, Cape Town, South Africa; ^2^Division of Infectious Diseases and Immunology, Department of Medicine, University of Massachusetts Medical School, Worcester, MA, United States; ^3^Institute of Infectious Disease and Molecular Medicine, Faculty of Health Science, University of Cape Town, Cape Town, South Africa

**Keywords:** West Nile virus, virus-like particle, envelope protein domain III, *Nicotiana benthamiana*, SpyTag/SpyCatcher

## Abstract

West Nile virus (WNV) is a globally disseminated *Flavivirus* that is associated with encephalitis outbreaks in humans and horses. The continuous global outbreaks of West Nile disease in the bird, human, and horse populations, with no preventative measures for humans, pose a major public health threat. The development of a vaccine that contributes to the “One Health” Initiative could be the answer to prevent the spread of the virus and control human and animal disease. The current commercially available veterinary vaccines are generally costly and most require high levels of biosafety for their manufacture. Consequently, we explored making a particulate vaccine candidate made transiently in plants as a more cost-effective and safer means of production. A WNV virus-like particle-display-based vaccine candidate was generated by the use of the SpyTag/SpyCatcher (ST/SC) conjugation system. The WNV envelope protein domain III (EDIII), which contains WNV-specific epitopes, was fused to and displayed on AP205 phage virus-like particles (VLPs) following the production of both separately in *Nicotiana benthamiana*. Co-purification of AP205 and EDIII genetically fused to ST and SC, respectively, resulted in the conjugated VLPs displaying EDIII with an average coupling efficiency of 51%. Subcutaneous immunisation of mice with 5 μg of purified AP205: EDIII VLPs elicited a potent IgG response to WNV EDIII. This study presents the potential plants being used as biofactories for making significant pharmaceutical products for the “One Health” Initiative and could be used to address the need for their local production in low- and middle-income countries (LMICs).

## Introduction

West Nile virus (WNV) is a member of the genus *Flavivirus* in the family *Flaviviridae* (Lindenbach et al., 2007). It is a zoonotic mosquito-borne virus that was first isolated in 1937 from the blood of a local woman in the West Nile district of Uganda (Smithburn et al., 1940). Since its first isolation, WNV has spread and become endemic in countries across Africa, the Americas, the Middle East, West Asia, and Australia (Castro-Jorge et al., 2019). Serological data have shown that many species can be infected by WNV, with the highest incidence of infection observed in birds, humans, and horses (Marfin et al., 2001). Infections in humans can result in febrile illness and less commonly neuroinvasive disease with significant mortality and morbidity (Gray and Webb, 2014). To date, disease treatment is supportive as there is no antiviral treatment and no available human vaccine. Approximately 20% of the infections in horses result in disease development, of which 90% involve neurological symptoms, with 30–40% fatality rates (Ward et al., 2006). As with humans, there is no antiviral treatment available for horses. However, several equine West Nile vaccines (whole inactivated virus, inactivated/live recombinant) produced in the United States (Castro-Jorge et al., 2019) and Europe (Rebollo et al., 2018) are licenced for use. These vaccines can be difficult and expensive to obtain in low- and middle-income countries (LMICs), with regards to import permits, cost of the vaccines, and the need for annual vaccination. Therefore, the preventative strategies tend to rely on the low-tech prevention of mosquito bites (Sule et al., 2018). The development of a vaccine that contributes to the “One Health” Initiative could be the answer to overcoming this challenge. The “One Health” Initiative is dedicated to improving the lives of all species, both human and animal, through the integration of human and veterinary medicine and environmental science.

The WNV genome is a positive-sense single-stranded RNA of ~11 kb that contains one open reading frame encoding a single polyprotein. This is co- and post-translationally processed into three structural proteins—the capsid, membrane (M, translated as prM, the precursor of the membrane protein), and envelope (E) proteins—and seven non-structural proteins (Chambers et al., 1990; Lindenbach et al., 2007). The E protein is the major virion surface protein and consists of three structurally distinct β-barrel envelope domains, namely, EDI, EDII, and EDIII. EDIII is an immunodominant domain that is highly variable among flaviviruses and has been suggested to contain the cell receptor binding sites for the infection (Mukhopadhyay et al., 2005; Zhang et al., 2017; Campos et al., 2018). Several studies using murine mAbs against E suggest that those directed against EDIII have the highest neutralising potency (Beasley and Barrett, 2002; Nybakken et al., 2005; Oliphant et al., 2005). Moreover, antibodies binding to EDIII do not result in antibody-dependent enhancement (ADE)—unlike antibodies binding to EDI and EDII—when inside cells expressing Fcγ receptors (Oliphant et al., 2006; Brandler and Tangy, 2013). Consequently, for the development of recombinant vaccines, EDIII has been the favoured target (Chu et al., 2007; Spohn et al., 2010; He et al., 2014).

Several antigen-display technologies have been developed and employed for different applications, such as protein cyclisation, creation of multi-component architectures such as hydrogels (Reddington and Howarth, 2015), vaccine development (Liu et al., 2014), and protein stabilisation for enzymes (Röder et al., 2017). Of interest for this study was the split-intein SpyTag/SpyCatcher (ST/SC) conjugation system. This is based on the spontaneous formation of irreversible isopeptide bonds between the complementary peptides. The ST and SC peptides originate from the Gram-positive bacterium *Streptococcus pyogenes*: the immunoglobulin-like collagen adhesion domain (CnaB2) of the fibronectin-binding protein of this bacterium contains an isopeptide bond between the amine of Lys31 and carbonyl carbon of Asp117. Consequently, the CnaB2 domain was split into two peptides: a 13 amino acid ST peptide containing the Asp117 residue and its complimentary SC peptide of 116 amino acids containing the Lys31 residue. The coupling of ST and SC by the formation of an amide bond has been demonstrated to occur at a range of temperatures (4–37°C) and pH values (5–8), in various buffers, and in the presence of non-ionic detergents (Zakeri et al., 2012).

Thrane et al. (2016) used the ST/SC technology to develop a Spy-virus-like particle (VLP) platform. This was achieved by the genetic incorporation of the ST or SC peptides at the N-terminus of the coat protein (CP) of the AP205 bacteriophage, yielding icosahedral particles that display 180 peptide-binding motifs on their surfaces. They evaluated the versatility of this platform by expressing 11 diverse vaccine candidate antigens and successfully expressed and coupled the Spy-antigens to the Spy-AP205 particles *in vitro*. The immunogenicity of the coupled products was evaluated in mice and robust IgG responses were elicited. This technology has also been used for the development of candidate vaccines for malaria (Brune et al., 2016; Janitzek et al., 2016; Singh et al., 2017; Yenkoidiok-Douti et al., 2019; Harmsen et al., 2020) and breast cancer (Palladini et al., 2018) that are predominantly produced in bacterial and insect cells. Janitzek et al. (2019) demonstrated the potential of this Spy-VLP system for the development of a combinatorial vaccine. The authors genetically fused the *L2* gene of human papillomavirus (HPV) and the SC peptide at the 5′ and 3′ ends of the AP205 *CP* gene, respectively, and the ST peptide to the N terminus of the VAR2CSA *Plasmodium falciparum* protein. These constructs were expressed in *Escherichia coli* and the purified products were coupled *in vitro* that resulted in the formation of AP205 VLPs, which displayed both the HPV L2 and VAR2CSA antigens at high density. High levels of anti-L2 and anti-VAR2CSA IgGs were elicited in vaccinated mice. With the recent coronavirus disease outbreak (COVID-19) (Zhu et al., 2020), several studies have been published using the ST/SC technology for the development of candidate vaccines. Tan et al. (2020) developed a SARS-CoV-2 candidate vaccine by displaying the spike receptor-binding domain (RBD) on the surface of the mi3 VLP computationally engineered from *Thermotoga maritima*. In another study, Zhang et al. (2020) displayed the SARS-CoV-2 spike trimer on the *Aquifex aeolicus* lumazine synthase (LuS) 60-mer nanoparticle scaffold also employing the ST/SC technology, with a coupling efficiency of ~91%. Mice immunised with the LuS:CoV-2 spike nanoparticle elicited a higher neutralising response than the mice immunised with the trimeric spike protein. Both these studies illustrated that a significantly lower dose of immunogen is required to elicit a potent neutralising response when the antigen is displayed on the surface of a particle, compared with when it is used as an immunogen on its own.

Since glycoproteins are generally difficult to produce in plants and are usually only produced at low yields, the display of these proteins on a particle may circumvent the requirement of high yields for vaccine doses for immunisation with a plant-produced vaccine. This technology offers a possibility for ease of candidate vaccine production in the instance of disease outbreaks and an opportunity for the development of multivalent vaccines. The Spy-VLP system, therefore, presents a favourable possibility for the development of candidate vaccines for flaviviruses, such as WNV, due to the potential of using EDIII as the display epitope.

In this study, we used the Spy-VLP system established by Thrane et al. (2016) adapted for the expression of ST-AP205 in *Nicotiana benthamiana*, as the display scaffold for SC-linked WNV EDIII. Both the tagged proteins were produced in plants: this technology has become popular in recent years due to the lower cost involved in biomass production and infrastructure required, in comparison with the cell-based expression systems (Twyman et al., 2003; Rybicki, 2009; Egelkrout et al., 2012; Martinez et al., 2012; Schillberg et al., 2019; Fischer and Buyel, 2020). Plant-produced ST-AP205 and SC-linked WNV-EDIII were purified, coupled, and their immunogenicity was assessed in mice.

## Materials and Methods

### Construction of Plant Expression Vectors

**Figure 1:**
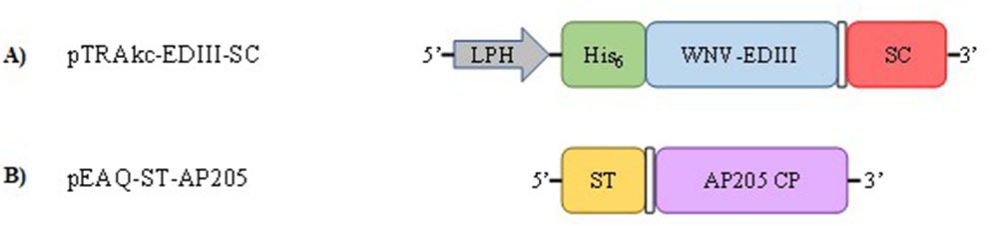
Schematic representation of plant expression constructs pTRAkc-EDIII-SC **(A)** and pEAQ-ST-AP205 **(B)**. The SpyCatcher (SC) peptide (red) is fused to the 3′ end of the West Nile virus–envelope protein domain III (WNV-EDIII) gene (blue) by a flexible linker; (GGGGS)_2_ (white box). The leader peptide derived from heavy chain (LPH) (murine mAB24 heavy chain) signal peptide allows for the translocation of WNV-EDIII-SC to the endoplasmic reticulum. The SpyTag (ST) peptide (orange) is fused to the 5′ end of AP205 coat protein (CP) gene (purple) by a flexible linker; (GGGGS)_2_ (white box).

The coding sequence of WNV prM-E from South African isolate SPU116/89 (GenBank accession number EF429197, amino acids 98-791) was codon-optimised for expression in *N. benthamiana* and synthesised by GenScript (GenScript Biotechnologies, Piscataway, NJ, USA). The *EDIII* domain was amplified from *prM-E* by PCR to generate a fragment with a hexa-histidine tag (His_6_) fused to the 5′ terminus and the SpyCatcher (SC) peptide sequence (GenBank accession number AFD50637, amino acids 65-129) joined to the 3′ terminus by a flexible linker (GGGGS)_2_ (Figure 1A; Supplementary Figure 1A). The generated fragment was ligated into the plant expression vector pTRAkc-ERH (Maclean et al., 2007) linearised with NcoI and XhoI using the In-Fusion® HD Cloning kit (Takara Bio, Saint-Germain-en-Laye, France) as per the instructions from the manufacturer resulting in pTRAkc-EDIII-SC. The recombinant pTRAkc-ERH plasmid was electroporated into *Agrobacterium tumefaciens* GV3101:pMP90RK cells (Maclean et al., 2007). pEAQ-ST-AP205 encoding Spytagged AP205 CP (Figure 1B; Supplementary Figure 1B), pEAQ-CRT and pEAQ-CNX encoding the human calreticulin (CRT), and calnexin (CNX) chaperones, respectively, have been described previously by Dennis (2019) and Margolin et al. (2020).

### Transient Expression in *N. benthamiana*

The 6-week-old *N. benthamiana* plants were vacuum infiltrated with either pEAQ-ST-AP205 (OD_600_ of 0.25) or pTRAkc-EDIII-SC (OD_600_ of 0.5), or co-infiltrated with pTRAkc-EDIII-SC and pEAQ-CRT/CNX (OD_600_ of 0.5 each = total OD_600_ of 1.0), or co-infiltrated with pEAQ-ST-AP205, pTRAkc-EDIII-SC, and pEAQ-CRT (OD_600_ of 0.3 each = total OD_600_ of 0.9) cultures with optical densities adjusted in infiltration medium [10 mM MgCl_2_, 10 mM MES (pH 5.6)]. A vacuum of ~100 kPa was applied to the chamber for infiltration of the leaves. As a negative control, a culture harbouring the pTRAkc-ERH vector lacking any gene cloned into the multiple cloning site (empty vector) was infiltrated at an OD_600_ of 0.25. The agroinfiltrated plants were returned to the plant growth room (temperature = 22°C, light:dark photoperiod = 16:8 h, relative humidity = 55%) and harvested at either 4, 5, or 6 days post infiltration (dpi).

### Extraction and Purification of EDIII-SC From *N. benthamiana* Leaves

The WNV-EDIII-SC proteins were extracted from leaves and purified as described by He et al. (2014) with some modifications. Leaves were harvested at 4 dpi and homogenised in 2 volumes of Tris buffer [100 mM Tris.HCl (pH 8.0), 150 mM NaCl, 1 × cOmplete™ EDTA-free protease inhibitor (Roche)] using an IKA® T-25 ULTRA-TURRAX® (Sigma-Aldrich, MO, USA) homogeniser. Homogenates were matured overnight (O/N) at 4°C with agitation and filtered through two layers of MiraCloth™ (Merck, Darmstadt, Germany). The filtered crude extract was clarified by centrifugation at 18,000 × g for 30 min at 4°C. The pH of the clarified extract was adjusted to 5.0 and the extract was then centrifuged at 18,000 × g for 30 min at 4°C and the supernatant recovered. The pH of the recovered supernatant was adjusted to 8.0 and subjected to another round of centrifugation. The supernatant was recovered, filtered through a 0.45 μM filter, and then loaded on a 5 ml nickel affinity column (HisTrap™ HP, GE Healthcare, IL, USA) and purified with an automated fast protein liquid chromatography system (ÄKTA Explorer™, GE-Healthcare). The eluted fractions were evaluated by Coomassie blue-stained sodium dodecyl sulphate–polyacrylamide gel electrophoresis (SDS-PAGE) and fractions showing a distinct 25 kDa WNV-EDIII-SC band were combined, concentrated, and buffer exchanged into Tris buffer using Amicon® Ultra-15 3K columns (Millipore®, MA, USA) according to the instructions from the manufacturer. The concentration of purified WNV-EDIII-SC proteins was determined by *DC*™ Protein Assay (Biorad, CA, USA) according to the instructions from the manufacturer using a standard bovine serum albumin curve.

### Extraction and Purification of Spytagged AP205 VLPs From *N. benthamiana* Leaves

Spytagged AP205 VLPs were extracted and purified by iodixanol density gradient ultracentrifugation as described by Dennis (2019).

### Coupling of ST- and SC-Linked Proteins

#### *In vitro* Coupling of Purified ST-AP205 VLPs and WNV-EDIII-SC Proteins

Purified ST-AP205 VLPs were coupled to the purified WNV-EDIII-SC proteins at 1:1 or 1:2 molar ratios O/N at 4°C. ST-AP205 VLPs and SC-linked EDIII antigens were mixed with 1 × cOmplete™ EDTA-free protease inhibitor (Roche, Basel, Switzerland) and coupling buffer [0.05 M MES, 0.05 M NaCl, 2.7 mM KCl, 10 mM NaH_2_PO_4_, 1.8 mM KH_2_PO_4_ (pH 6.4)] and evaluated for coupling by Coomassie blue-stained SDS-PAGE.

#### Co-expression of ST-AP205 and WNV-EDIII-SC/CRT

Cultures harbouring pEAQ-ST-AP205, pTRAkc-EDIII-SC, and pEAQ-CRT were co-infiltrated at OD_600_ 0.3 each (total OD_600_ = 0.9). The infiltrated leaves were harvested at 5 dpi and AP205:EDIII VLPs purified as described in Extraction and purification of Spytagged AP205 VLPs from *N. benthamiana* leaves. The presence of coupled products was evaluated by western blot with polyclonal rabbit anti-ST-AP205 and polyclonal rabbit anti-WNV-EDIII sera.

#### Co-extraction and Purification of ST-AP205- and WNV-EDIII-SC-Infiltrated Leaves

Leaves individually infiltrated with ST-AP205 and WNV-EDIII-SC/CRT were harvested at 5 dpi and combined in a 1:2 fresh leaf weight (FLW) ratio and co-extracted. They were homogenised in two volumes of 1× PBS (pH 7.4) using a Moulinex™ juice extractor and the leaf pulp was incubated with the extracted juice at 4°C O/N with agitation to allow for coupling. AP205:EDIII VLPs were purified by density gradient ultracentrifugation as described in Extraction and purification of Spytagged AP205 VLPs from *N. benthamiana* leaves. The fractions of 1 mL were collected from the bottom of the tube and fractions 3–5 (29% iodixanol) were combined and filtered through a 0.22 μM filter. As a negative control, the leaves harvested from plants infiltrated with pTRAkc-ERH (empty vector) were treated the same as described above.

### Western Blot Analysis

Protein expression and coupling were analysed by 12.5% SDS-PAGE and proteins were blotted on a nitrocellulose membrane using the semi-dry blotting system (BioRad). The membranes were probed with antibodies diluted in blocking buffer [1× PBS (pH 7.4), 0.1% Tween-20, 5% fat-free milk]. WNV-EDIII-SC and ST-AP205 proteins were probed using protein-specific in-house produced rabbit antisera (diluted 1:20,000) against WNV-EDIII and Spytagged AP205 CP, respectively. The proteins were detected with alkaline phosphatase-conjugated polyclonal goat anti-rabbit antibody (Biocom Africa) and the signal was visualised with 5-bromo-4-chloro-3-indolyl phosphate (BCIP) and nitroblue tetrazolium (NBT) phosphatase substrate (BCIP/NBT 1-component, KPL).

The yield of coupled AP205:EDIII was determined by densitometric analysis of anti-WNV-EDIII western blots using GeneTools software (Synoptics Inc., MD, USA) with an *E. coli* produced WNV-EDIII protein standard. The coupling efficiencies were estimated by densitometric analysis of bands visualised on the anti-ST-AP205 western blots. The intensity value of the AP205:EDIII complex protein band (41.5 kDa) was divided by the intensity value of the ST-AP205 protein band (16.5 kDa) and multiplied by 100 to estimate the percentage coupling efficiency. The antigen-display capacity (number antigens/VLP) was estimated by multiplying the coupling efficiency by 180.

### Transmission Electron Microscopy (TEM)

Glow-discharged carbon-coated copper grids (mesh size 200) were floated on 20 μl of purified sample for 3 min and washed five times with sterile water. The samples were negatively stained for 1 min with 2% w/v uranyl acetate and viewed using a Tecnai G2 transmission electron microscope.

### Immunisation of Mice

All animal work was approved by the Faculty of Health Sciences Animal Ethics Committee, University of Cape Town (FHS AEC ref no: 019_034). Female BALB/c mice of 6- to 8-week-old were divided into three groups (*n* = 6 per group). Group 1 received a mock antigen of 100 μl purified negative plant protein extract from plants infiltrated with an empty pTRAkc-ERH vector with 5% Montanide Gel adjuvant (Seppic, Courbevoie, France). Groups 2 and 3 received 5 μg of purified plant-produced AP205:EDIII VLPs (produced by co-extraction) and soluble WNV-EDIII, respectively, with 5% Montanide Gel adjuvant. Prior to the study, 100 μl of blood (pre-immunised serum) was drawn from each mouse (*n* = 18). Inocula were administered by subcutaneous injection on days 0, 14, and 35 with the same dosage. Mice were euthanised on day 40 and blood (immunised serum) was collected by cardiac puncture.

### Indirect ELISA Detection of Anti-EDIII Antibodies in Mouse Sera

The WNV-EDIII was produced in *E. coli* from a pColdTF-WNV-EDIII construct made previously and purified using the BugBuster™ Protein Extraction kit (Merck, Darmstadt, Germany) according to instructions from the manufacturer. Enzyme linked immunosorbant assay (ELISA) plates were coated with 100 ng *E. coli*-produced WNV-EDIII diluted in coating buffer [10 mM Tris (pH 8.5)] and incubated O/N at 4°C. To determine the anti-EDIII response elicited, individual mouse sera (*n* = 6) for each immunisation group and mouse sera (*n* = 6 per group) pooled into vaccine groups (*n* = 3) were analysed. Pooled, pre-immunised, and immunised mouse sera for each immunisation group and individual immunised mouse sera from each immunisation group were diluted 1:1,000 in TBS [3% BSA in 1x TBS (50 mM Tris, 150 mM NaCl, pH 7.5)] and 100 μl added to each well and the plate incubated for 1 h at 37°C. Blank wells with no antibodies were included as a background control. Bound antibodies were detected by alkaline phosphatase-conjugated goat anti-mouse-IgG (Sigma, MO, USA) and subsequent addition of SIGMAFAST™ p-Nitrophenyl phosphate substrate (Sigma) and the absorbance measured at 405 nm. To determine the anti-EDIII binding titres of each group, individual immunised mouse sera (*n* = 6 per group) were diluted in TBS in a 4-fold series ranging from 1:500 to 1:128,000. Sera from the mice vaccinated with the negative plant protein extract (empty pTRAkc-ERH) served as a negative control. The ELISAs were repeated three times independently with each sample analysed three times.

The statistical analyses were performed using GraphPad Prism software version 9.2 (GraphPad, CA, USA). Comparisons between the pre-immunised and immunised serum of vaccination groups or between the immunised sera of vaccination groups were performed by the Student's two-tailed *t*-test. A *p* < 0.05 indicated a statistically significant difference.

## Results

### Expression and Purification of SC-Linked WNV EDIII From Plants

**Figure 2:**
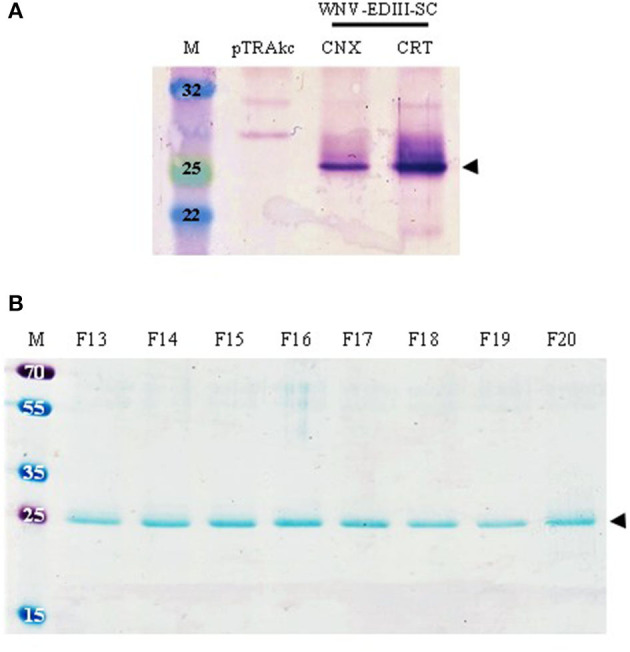
The expression and purification of plant-produced WNV-EDIII-SC (25 kDa). **(A)** Immunoblot of crude leaf extracts (4 dpi) of WNV-EDIII-SC co-infiltrated with either CNX or CRT. Lanes were loaded with 25 μg TSP each for direct comparison and WNV-EDIII-SC protein was detected with in-house-produced anti-WNV-EDIII serum. **(B)** Coomassie blue-stained SDS-PAGE of eluted fractions of WNV-EDIII-SC following pH precipitation and nickel-affinity chromatography. pTRAkc: pTRAkc-ERH empty plant-expression vector. CNX, calnexin; CRT, calreticulin; F, fraction.

Expression of WNV-EDIII-SC in leaves using varying concentrations (OD_600_ 0.25 vs. 0.5) of agroinfiltration cultures, and harvesting leaves at 4, 5, and 6 dpi, was assessed. The crude leaf extracts were evaluated by anti-WNV-EDIII western blotting and no protein band of the expected size (25 kDa) was observed at all dpi tested. However, when genes for human chaperone proteins CNX or CRT were co-infiltrated—these are known to increase the yield of viral glycoproteins (Margolin et al., 2020)—the expected 25 kDa band was observed (Figure 2A—black arrow). This was not seen in the negative control lane (pTRAkc). A higher yield of soluble WNV-EDIII-SC was observed in leaves co-infiltrated with CRT in comparison to CNX as assessed by the intensity of the protein band obtained at 4 dpi (optimal dpi selected based on the previous optimisation experiments). Accordingly, the production of WNV-EDIII-SC was scaled up by co-infiltrating leaves with the CRT chaperone.

**Figure 3:**
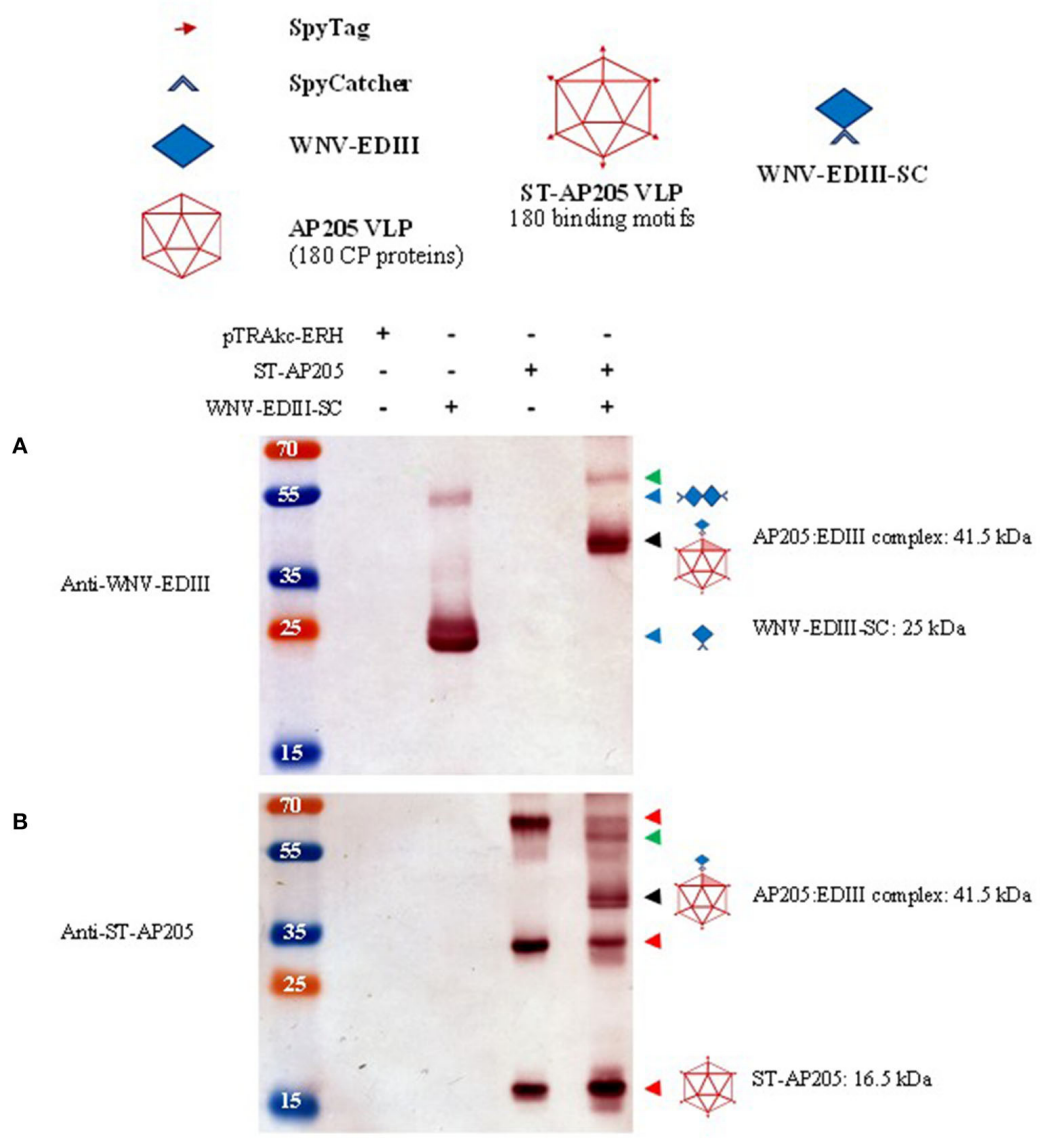
Analysis of ST-AP205 and EDIII-SC coupling. Schematic diagram of coupling components: each AP205 CP has an ST peptide (16.5 kDa) and 180 CP proteins make up the icosahedral (*T* = 3) ST-AP205 virus-like particles (VLP) and as a result, there are 180 binding motifs that can couple to WNV-EDIII-SC (25 kDa). Western blot analysis of complex formation visualised with rabbit anti-WNV-EDIII antiserum **(A)** or rabbit anti-ST-AP205 antiserum **(B)**. Successful coupling is indicated by a molecular weight shift to 41.5 kDa (monomer—shaded triangle, ~58 kDa coupled dimer—AP205 CP dimer with one coupled WNV-EDIII protein) only in co-purified extracts containing ST-AP205 and WNV-EDIII-SC. pTRAkc-ERH: empty plant-expression vector. AP205: *Acinetobacter* bacteriophage AP205 CP. EDIII: West Nile virus (WNV) envelope domain III. ST, SpyTag; SC, SpyCatcher. Red arrows: ST-AP205 protein subunits (16.5 kDa monomer, 33 kDa dimer, 66 kDa tetramer). Blue arrows: WNV-EDIII-SC protein subunits (25 kDa monomer, ~50 kDa dimer). Black arrow: 41.5 kDa AP205:EDIII coupled complex monomer. Green arrow: ~58 kDa AP205:EDIII coupled complex dimer.

Following large-scale infiltration of plants, WNV-EDIII-SC protein was successfully purified by pH precipitation and subsequent nickel-affinity chromatography. Eluted fractions were evaluated by Coomassie blue-stained SDS-PAGE for the presence of WNV-EDIII-SC protein (Figure 2B—black arrow), and fractions 13–20 were pooled and quantitated after simultaneous concentration and buffer exchange to remove imidazole. The yield of purified WNV-EDIII-SC protein from several experiments was calculated to be in the range of 33.5–69 mg/kg FLW. Western blot analysis of the quantified WNV-EDIII-SC protein also showed the presence of a dimerisation product of approximately 50 kDa in addition to the 25 kDa monomer (Figure 3A—blue arrows) which was not visible on the Coomassie-stained gel (Figure 2B).

### *In vitro* Coupling of Purified ST-AP205 VLPs and WNV-EDIII-SC Proteins

The ST-AP205 VLPs were extracted from leaves and their expression was confirmed by the presence of a 16.5 kDa band by use of anti-ST-AP205 serum in a western blot (Figure 3B—red arrows). In addition to the monomer, a 33 kDa dimer and 66 kDa tetramer of ST-AP205 CP were also observed. Particle integrity was confirmed by TEM (Supplementary Figure 2).

Purified ST-AP205 VLPs and WNV-EDIII-SC proteins were coupled at two molar ratios−1:1 and 1:2, respectively—and successful coupling was evaluated by SDS-PAGE. A successful coupling was evidenced by a molecular weight shift to the expected size of 41.5 kDa (Supplementary Figure 3), representing one ST-AP205 CP subunit coupling to one WNV-EDIII-SC protein. Uncoupled ST-AP205 CP (16.5 kDa) and WNV-EDIII-SC (25 kDa) were also observed in both molar coupling reactions. In the 1:2 molar ratio reaction, a more intense band of WNV-EDIII-SC was observed than in the 1:1 molar ratio reaction; however, the intensity of the coupled AP205:EDIII band was similar for both ratios.

### Co-expression of ST-AP205 and WNV-EDIII-SC/CRT

Following the success of *in vitro* coupling of purified plant-produced WNV-EDIII-SC proteins to ST-AP205 VLPs, *in vivo* coupling by co-expression of these constructs in *N. benthamiana* was investigated. The leaves were infiltrated with cultures harbouring pEAQ-ST-AP205, pTRAkc-EDIII-SC, and pEAQ-CRT, and harvested at 5 dpi based on the previous optimisation. AP205:EDIII VLPs were purified and collected fractions were analysed by anti-ST-AP205 western blot (Supplementary Figure 4). Protein bands of uncoupled ST-AP205 CP subunits (monomer, dimer, and tetramer) and the coupled 41.5 kDa AP205:EDIII complex were observed. Additionally, an approximately 58 kDa-sized band was detected—most likely representing an AP205:EDIII coupled dimer consisting of two AP205 CPs (33 kDa) with only one coupled WNV-EDIII protein (25 kDa). In fractions, 6–8 (29% iodixanol) protein doublets were observed for the uncoupled ST-AP205 CP subunits, which could be a result of protein degradation or loss of the ST peptide by enzymatic cleavage during purification or storage at 4°C (AP205 CP only ~14 kDa).

### Co-extraction and Purification of ST-AP205 and WNV-EDIII-SC Infiltrated Leaves

Having confirmed the expression of ST-AP205 VLPs and WNV-EDIII-SC proteins in *N. benthamiana* infiltrated using optimal conditions, the leaves from plants individually infiltrated with ST-AP205 and WNV-EDIII-SC/CRT were combined and the tagged VLPs and proteins co-extracted to facilitate potential coupling of WNV-EDIII-SC to ST-AP205 VLPs prior to the purification on a density gradient. Following density gradient ultracentrifugation, 1 ml fractions were collected from the bottom of the tube and the AP205:EDIII-containing fractions combined. Western blot analysis of the combined sample revealed the expected molecular weight shift of AP205:EDIII to 41.5 kDa when probed with either anti-WNV-EDIII or anti-ST-AP205 antibodies (Figures 3A,B—black arrows). In addition, a ~58 kDa-sized band representing a dimer of the AP205 CP with one WNV-EDIII protein coupled to it was observed (Figures 3A,B—green arrows). Similar to the *in vitro* and *in vivo* coupling reactions, the protein bands of uncoupled ST-AP205 CP subunits (monomer, dimer, and tetramer) were observed (Figure 3B—red arrows) but unlike for the *in vitro* coupling reaction, no uncoupled WNV-EdIII-SC protein was observed. As expected, there were no bands visible in the sample fractions purified similarly from the leaves infiltrated with empty pTRAkc-ERH.

**Figure 4:**
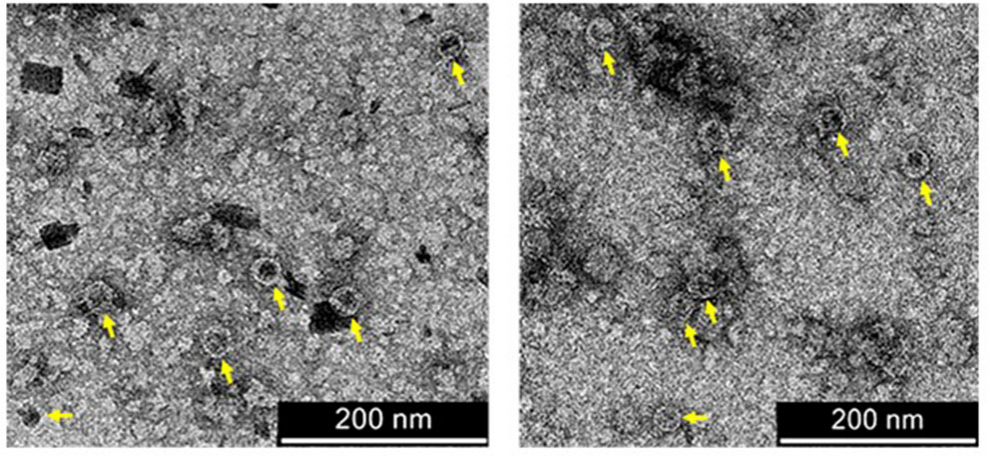
Transmission electron micrographs of purified coupled VLPs. AP205:EDIII VLPs of 30 nm in diameter are indicated by yellow arrows. Scale bars = 200 nm.

Since the co-extraction method yielded higher amounts of AP205:EDIII VLPs than the co-infiltration method (Supplementary Figure 5), co-extracted VLPs were further analysed. The integrity of the AP205:EDIII VLPs was assessed by TEM of filtered samples containing AP205:EDIII. The particles with a diameter of 30 nm were observed (Figure 4—yellow arrows). The concentration of the final purified AP205:EDIII complex was determined by anti-WNV-EDIII western blot to be 90 μg/ml (Supplementary Figure 6) with a total yield of ~10.5 mg/kg FLW. On average, the coupling efficiency calculated from repeat experiments was determined to be 51%, translating to ~92 WNV-EDIII antigens displayed per AP205 particle. However, the coupling efficiency calculation does not take into account the uncoupled dimer and tetramer ST-AP205 CP bands (Figure 3B—red arrows) and the intensity of these bands together with that of the monomeric band suggests that the coupling efficiency of this specific experiment was overestimated. The complete absence of uncoupled WNV-EDIII-SC bands (Figure 3A) suggests that the low coupling efficiency may be due to the saturation of the protein during coupling. To address this, we evaluated several co-extraction ratios of leaves infiltrated with ST-AP205 to leaves infiltrated with WNV-EDIII-SC/CRT and found that the increase in FLW of the latter resulted in a decrease in uncoupled ST-AP205 CP subunits, suggesting an increase in the coupling efficiency (Supplementary Figure 7). However, with an increase in WNV-EDIII-SC FLW, we observed a decrease in AP205:EDIII complex yield which is why an extraction ratio of 1:2 was used for generating AP205:EDIII VLPs for testing their immunogenicity in mice.

### Immunogenicity of Plant-Produced AP205:EDIII and WNV-EDIII

**Figure 5:**
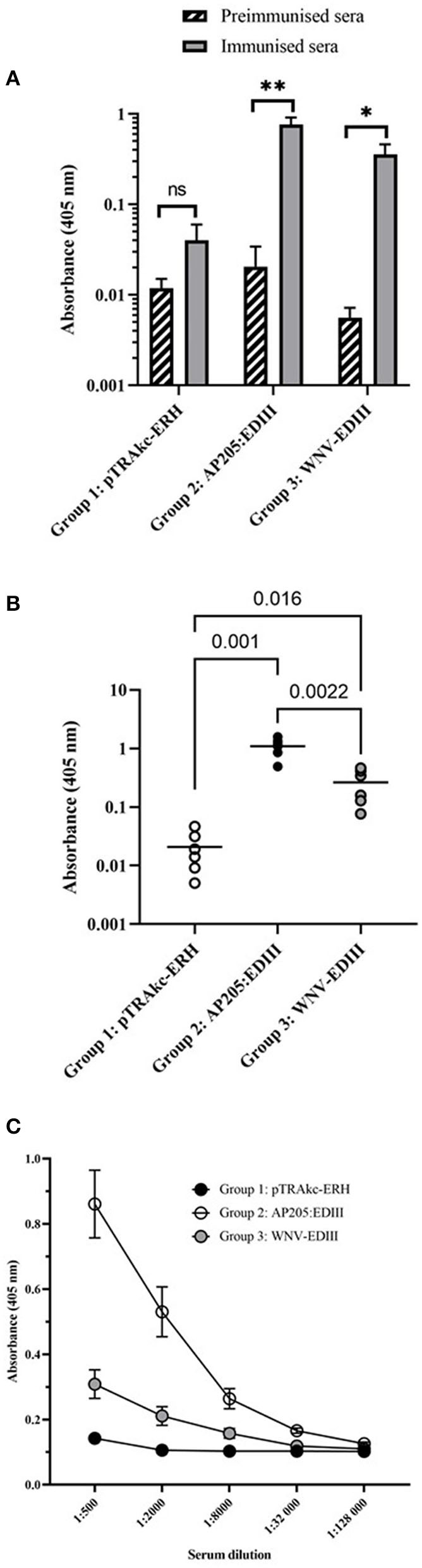
Indirect ELISAs of mouse sera using *Escherichia coli* produced WNV-EDIII as antigen. **(A)** Antibody responses before and after immunisation of vaccine groups. Pooled mouse sera (*n* = 6 per group) for each vaccine group, pre-immunised and immunised bleed was diluted to 1:1,000 and evaluated for the presence of anti-WNV-EDIII antibodies. Bars represent the mean ± SEM from three independent experiments. The *y*-axis shows the log10 absorbance readings at 405 nm. **(B)** Comparison of mouse antibody responses post-immunisation of individual mouse sera (*n* = 6) for each vaccine group as tested at the dilution 1:1,000. Shown are the individual absorbance readings and group means (log10 absorbance at 405 nm). The student's *t*-test was used to determine the significance levels (*p*-values are indicated between groups). **(C)** A 4-fold titration of mouse antiserum (*n* = 6) from 1:500 to 1:128,000 produced against pTRAkc-ERH negative control, AP205 VLPs displaying WNV-EDIII and soluble WNV-EDIII vaccine candidates. The mean ± SEM of three independent experiments with technical triplicates for each group is presented. NS, * and ** indicate *p* values > 0.05, < 0.05, and < 0.01, respectively.

To evaluate the immunogenicity of plant-produced AP205 VLPs displaying WNV-EDIII prepared by co-extraction and soluble WNV-EDIII protein, BALB/c mice were immunised with three doses of 5 μg each and serum tested for WNV-EDIII-specific antibodies. Individual mouse sera of each vaccine group was pooled for the pre-immunised and immunised bleeds and the presence of WNV-EDIII antibodies evaluated by indirect ELISA using *E. coli* produced WNV-EDIII as antigen. Sera from mice immunised with either AP205:EDIII VLPs or soluble WNV-EDIII both had a statistically significant higher absorbance value, *p* = 0.007 and *p* = 0.02, respectively, compared with their respective pre-immunised sera (Figure 5A). This suggests the induction of potent anti-WNV-EDIII responses. The negative control serum—mock antigen from purified leaf material infiltrated with empty pTRAkc-ERH vector—did not have a statistically significant difference in absorbance between the pre-immunised and immunised sera (*p* = 0.2).

To analyse the difference in antibody responses between the three vaccination groups, immunised sera from individual mice (*n* = 6) for each group were diluted to 1:1,000 and evaluated by indirect ELISA against *E. coli*-produced WNV-EDIII. Both the candidate vaccine groups of mice immunised with AP205:EDIII VLPs and soluble WNV-EDIII had higher absorbance values (*p* = 0.001 and *p* = 0.016, respectively) in comparison with the negative control group (Figure 5B). In addition, mice immunised with AP205:EDIII VLPs had a significantly better response (*p* = 0.0022) than mice immunised with the soluble WNV-EDIII immunogen. The antibody endpoint titre for the vaccination groups was determined by a 4-fold titration of immunised sera from individual mice (Figure 5C). The AP205:EDIII VLP group had an endpoint binding titre of 1:32,000 while the soluble WNV-EDIII group had an endpoint binding titre of 1:8,000.

## Discussion

The use of antigen-display technology for the production of particulate candidate vaccines has become popular in the last few years (Spohn et al., 2010; Chen et al., 2011; Brune et al., 2016; Janitzek et al., 2016; Singh et al., 2017; Palladini et al., 2018; Yenkoidiok-Douti et al., 2019; Harmsen et al., 2020). The advantages of using VLPs instead of soluble subunit antigens as vaccines are in large part due to their size (20–200 nm), which allows for optimal uptake by antigen-presenting cells and their dense repetitive surface structures that enable complement fixation and B cell receptor clustering, responsible for the activation of the innate immune system and antibody production (Chen and Lai, 2013; Thrane et al., 2016).

The immunodominant domain III of the WNV envelope protein (EDIII) was selected for display on AP205 particles, both to reduce the possibility of ADE seen with the complete protein, and to enhance its immunogenicity. Generally, viral proteins accumulate at reasonable levels when expressed in plants; however, viral glycoproteins express poorly and often below the threshold of detection in plants, for reasons not yet fully understood. Our laboratory has, however, succeeded in dramatically increasing the expression of a variety of complex virus-derived envelope glycoproteins, by co-expression of these with one or other of the human ER chaperone proteins, such as CRT and CNX. Margolin et al. found that plant-expressed ER-targeted glycoproteins from Epstein–Barr virus, Rift Valley fever virus, chikungunya virus, and human immunodeficiency virus were only detectable when co-expressed with a human chaperone protein (Margolin et al., 2020). It is suggested these ensure correct folding, thus minimising degradation of proteins by targeting them to the ER-associated degradation (ERAD) pathway.

This study describes the expression of WNV-EDIII with the SC peptide fused to its C terminus. The SC rather than the ST peptide was selected for genetic fusion to the *EDIII* gene because of the increased expression yields observed for several *E. coli*-produced proteins when fused to the SC compared with the ST peptide (Thrane et al., 2016). Additionally, our lab has demonstrated that a higher yield of AP205 CP is obtained from infiltrated *N. benthamiana*, when it is fused to the ST rather than to the SC peptide (Dennis, 2019). Infiltration of WNV-EDIII-SC in *N. benthamiana* resulted in no detectable WNV-EDIII-SC protein expression. Subsequently, we co-expressed WNV-EDIII-SC with the human chaperone proteins, CNX and CRT. The co-expression of WNV-EDIII-SC with either human chaperone allowed detectable levels of WNV-EDIII-SC as assessed by anti-EDIII western blot. A higher yield of protein was obtained when WNV-EDIII-SC was co-expressed with CRT, which was expected as CRT is a soluble luminal protein situated within the ER that is known to associate with soluble proteins, whereas CNX is known to associate with membrane-bound proteins (Ellgaard and Helenius, 2003). In this study, we showed that co-expression of WNV-EDIII-SC with CRT has resulted in yields of up to 69 mg/kg FLW that are similar to those reported by He et al. (2014).

Several studies have focused on the molar coupling of purified ST-and SC-linked components, which can be a time-consuming and expensive process (Brune et al., 2016; Thrane et al., 2016; Singh et al., 2017; Palladini et al., 2018; Yenkoidiok-Douti et al., 2019; Harmsen et al., 2020). Other previous studies conducted *in planta* have looked at by-passing this by either co-infiltration (Peyret et al., 2020) or by co-extraction of leaves separately exposed either by infection or infiltration (Röder et al., 2017) of the ST- and SC-linked components. We investigated each of these methods and were able to successfully couple WNV-EDIII-SC to ST-AP205 VLPs for all three tested groups. The ease of coupling by either co-expression or co-extraction compared to the more laborious process of carrying out the various purification methods required for *in vitro* coupling was viewed as a more promising coupling model for commercialisation purposes, specifically with regard to scaled-up production. We observed that the co-extraction of leaves from plants infiltrated separately with ST-AP205 and WNV-EDIII-SC/CRT resulted in a higher yield of AP205:EDIII VLPs in comparison with the yield when these constructs were co-infiltrated.

The formation of a stable isopeptide bond from co-extracts containing ST-AP205 and WNV-EDIII-SC was confirmed by western blot with a mobility shift to 41.5 kDa, and the assembly of VLPs confirmed by TEM. In addition to the monomeric coupled complex of AP205:EDIII, a protein band of ~58 kDa was detected by both anti-WNV-EDIII and anti-ST-AP205 western blots. We proposed that this band is a coupled dimeric product that consists of two AP205 CP (33 kDa) with only one coupled WNV-EDIII (25 kDa): given that we continuously observed a dimer and a tetramer of uncoupled ST-AP205 CP in addition to a coupling efficiency of only 51%, it is highly probable that this could be the case. Covalent cross-linking of proteins is a common phenomenon of plant expression (Castells-Graells and Lomonossoff, 2021). Castells-Graells and Lomonossoff showed that the insect virus, *Nudaurelia capensis* omega virus α-peptide formed multimers only when in the presence of plant material since procapsids produced in insect cells only consisted of monomeric α-peptide (Castells-Graells and Lomonossoff, 2021). However, when these procapsids were incubated in plant extracts, α-peptide dimers were observed similar to those when the procapsids were produced in *N. bethamiana*.

Despite the coupling efficiency being half of what it could potentially be—possibly due to steric hindrance preventing EDIII coupling to every CP subunit since it is larger and we expect that we are grossly overestimating the coupling efficiency of the purified sample used for immunisation—the plant-produced AP205:EDIII VLPs elicited a potent immune response against WNV-EDIII in BALB/c mice, with an antibody titre of 1:32,000. Additionally, the IgG antibody level elicited by AP205:EDIII VLPs was significantly higher than that of soluble WNV-EDIII. We anticipated that the VLP-based candidate vaccine would induce a stronger immune response than the soluble WNV-EDIII due to the enhanced antibody responses observed against WNV-EDIII chemically conjugated to VLPs (Spohn et al., 2010; Cielens et al., 2014). The low immunogenicity of soluble protein antigens can be ascribed to their small size (<10 nm), their increased susceptibility to proteolytic degradation, and poor capacity to activate the innate immune system (Thrane et al., 2016), therefore a higher dose, multiple injections, and strong adjuvants are required to ensure a strong immune response (He et al., 2014).

Encouragingly, a significantly better WNV-EDIII response was elicited by mice immunised with AP205:EDIII VLPs at the same dose as mice immunised with soluble WNV-EDIII. However, the 5 μg dose of AP205:EDIII only represents ~1.54 μg of WNV-EDIII protein due to a coupling efficiency of only 51%, which we expect is overestimated due to the significant amount of uncoupled AP205 CP subunits observed by western blot; thus stimulating a superior immune response with less WNV-EDIII immunogen than the soluble WNV-EDIII. The same effect was observed for the production of ST/SC coupled SARS-CoV-2 candidate vaccines, where a lower dose of immunogen coupled to a particle elicited a better immune response in comparison with the corresponding soluble protein on its own (Tan et al., 2020; Zhang et al., 2020). These findings illustrate that a significantly lower dose of immunogen is required to elicit a potent immune response when the antigen is displayed on the surface of a particle, compared with the dose when it is used as an immunogen on its own.

Interestingly, similar antibody titres to the soluble WNV-EDIII were observed in studies reported from a different laboratory where mice were immunised with 25 μg of either plant-produced soluble WNV-EDIII (He et al., 2014; Lai et al., 2018), or WNV-EDIII displayed on the surface of HBcAg (He et al., 2021). Their immunisation dose was five times more than what was used in our study, however, we observed similar or superior antibody binding titres with our lower dose of soluble WNV-EDIII and AP205:EDIII VLPs, respectively. Additionally, immunisation of 5 μg plant-made AP205:EDIII VLPs or soluble WNV-EDIII was observed to stimulate an immune response of similar potency to *E. coli*-produced WNV-EDIII immunised at 25 μg (He et al., 2014). This suggests that plants are just as effective at producing WNV-EDIII proteins as *E. coli*.

The antibody responses we observed for both soluble WNV-EDIII and AP205:EDIII VLPs have the potential to be neutralising and/or protective. Lai et al. have previously demonstrated that plant-made WNV-EDIII elicited potent neutralising activities against WNV and protected mice against lethal WNV challenge (Lai et al., 2018). The authors also showed that their plant-produced WNV-EDIII immunogen did not result in ADE of either Zika virus or dengue virus infection.

In a recent study by He et al. HBcAg-WNV-EDIII VLPs were produced in *N. benthamiana* at a yield of 1.2 g/kg FLW (He et al., 2021). In this study, we report a yield of ~10 mg/kg FLW and from previous experiments, we have obtained a yield of purified AP205:EDIII VLPs of ~36 mg/kg FLW. Further optimisation of our purification procedure is required to obtain similar yields as reported by He et al. as required for vaccine manufacturing. However, our results show that a much lower dose of the AP205:EDIII VLPs was able to induce a higher immune response than reported in their study, which might alleviate the need for increased yields.

In this study, we have shown an apparent advantage of EDIII display compared with soluble subunit EDIII to be used as a vaccine in terms of serum antibody titres obtained in vaccinated mice, cell-mediated responses may also be significantly enhanced, as it is well-known for VLP-based vaccines (Rybicki, 2019). Although the results observed here are promising, future neutralisation experiments, assays of cell-mediated responses, and WNV challenge studies in mice are required to confirm that these vaccine candidates can protect against WNV infection.

### Conclusion

The global distribution of WNV, its association with severe neurological disease in its avian reservoir hosts and dead-end mammalian hosts, lack of antiviral treatment, and in the case of humans, lack of a licenced vaccine, demand the development of an affordable and effective vaccine. With little to no infrastructure for the development of these therapeutics in developing countries, the high costs associated with the importation of these products and the difficulties associated with the regulatory procedures for the procurement of permits further necessitate the local development of these products.

In this study, we demonstrate that a transient plant expression system provided a rapid method for the production of both WNV-EDIII and AP205 VLPs in *N. benthamiana* and that the use of the ST/SC technology allowed for ease of display of the WNV-EDIII on the AP205 particle surface. Both AP205:EDIII VLPs and soluble WNV-EDIII elicited potent humoral responses in mice, equivalent to that of protective WNV-EDIII antigens in previous studies. These outcomes show that the ST/SC technology and plant molecular farming have the potential for creating opportunities for LMICs to locally produce their pharmaceuticals, thus working towards fulfilling the goals of the “One Health” Initiative. It also indicates the feasibility of making poly-flavivirus vaccines, by coupling SC-tagged EDIII domains of a variety of flaviviruses to a common carrier, such as ST-coupled AP205—and of making the whole system in plants.

## Data Availability Statement

The raw data supporting the conclusions of this article will be made available by the authors, without undue reservation.

## Author Contributions

ER conceived the project. JS, AM, and AC designed the study and developed the work plan. JS performed the experiments and drafted the manuscript. All the authors edited, read, and approved the final manuscript.

## Funding

We thank the Poliomyelitis Research Foundation (PRF) for financial support of the project (19/21), the PRF, Council for Scientific and Industrial Research (CSIR), Biopharming Research Unit (BRU), the Postgraduate Publication Incentive (PPI) of UCT, and the Carnegie Corporation for financial support to JS. This publication was made possible in part by a grant from Carnegie Corporation of New York. The statements made and views expressed are solely the responsibility of the authors.

## Conflict of Interest

The authors declare that the research was conducted in the absence of any commercial or financial relationships that could be construed as a potential conflict of interest.

## Publisher's Note

All claims expressed in this article are solely those of the authors and do not necessarily represent those of their affiliated organizations, or those of the publisher, the editors and the reviewers. Any product that may be evaluated in this article, or claim that may be made by its manufacturer, is not guaranteed or endorsed by the publisher.
