# Supplementary material for: A Plant-Produced Virus-Like Particle Displaying Envelope Protein Domain III Elicits an Immune Response Against West Nile Virus in Mice
Source: Front Plant Sci. 2021 Sep 13;12:738619. doi: 10.3389/fpls.2021.738619 (PMC8475786; doi:10.3389/fpls.2021.738619)
Supplement: Supplementary file 1 [file Data_Sheet_1.DOCX]

Supplementary Material

**A)**


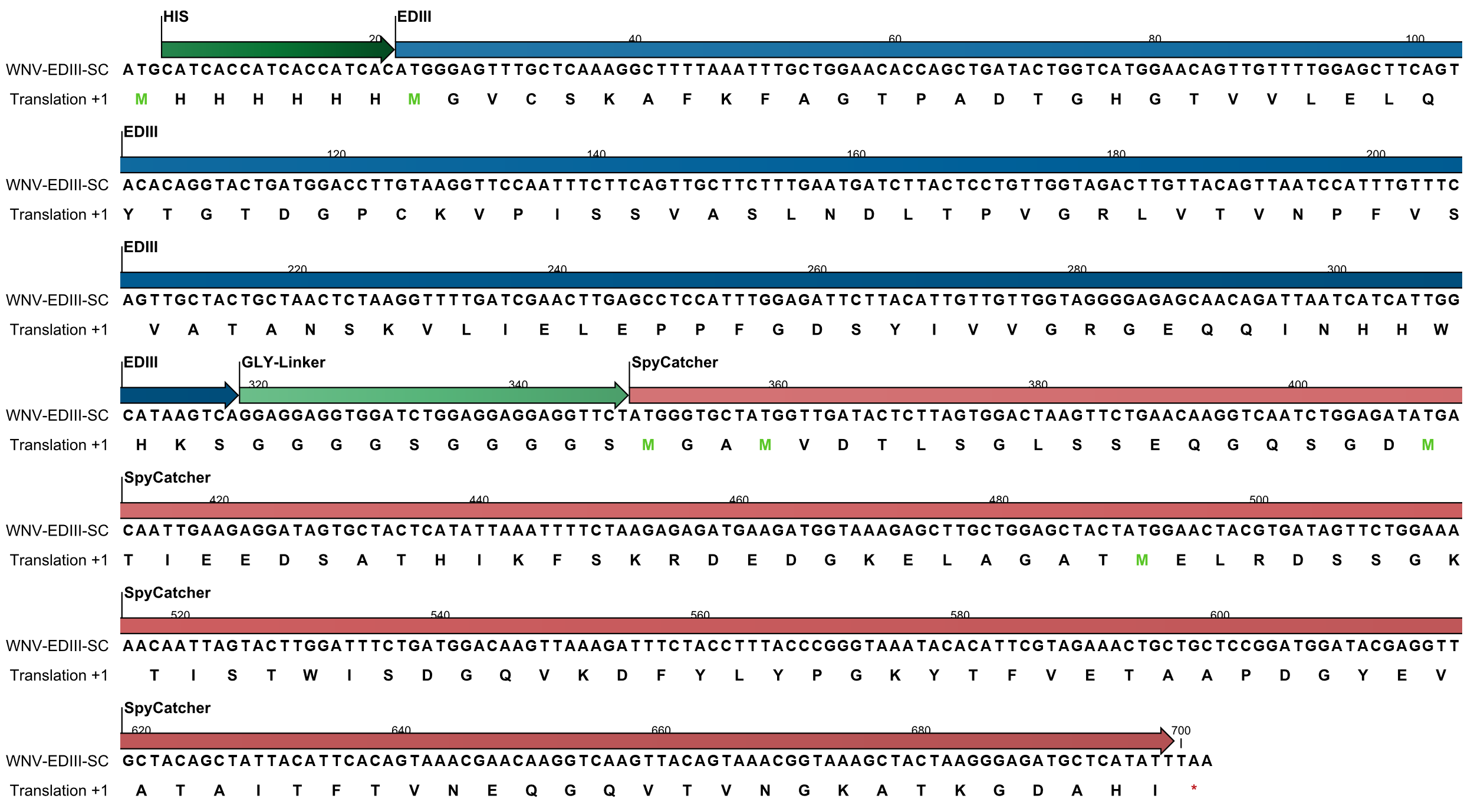


5’ATGCATCACCATCACCATCACATGGGAGTTTGCTCAAAGGCTTTTAAATTTGCTGGAACACCAGCTGATACTGGTCATGGAACAGTTGTTTTGGAGCTTCAGTACACAGGTACTGATGGACCTTGTAAGGTTCCAATTTCTTCAGTTGCTTCTTTGAATGATCTTACTCCTGTTGGTAGACTTGTTACAGTTAATCCATTTGTTTCAGTTGCTACTGCTAACTCTAAGGTTTTGATCGAACTTGAGCCTCCATTTGGAGATTCTTACATTGTTGTTGGTAGGGGAGAGCAACAGATTAATCATCATTGGCATAAGTCAGGAGGAGGTGGATCTGGAGGAGGAGGTTCTATGGGTGCTATGGTTGATACTCTTAGTGGACTAAGTTCTGAACAAGGTCAATCTGGAGATATGACAATTGAAGAGGATAGTGCTACTCATATTAAATTTTCTAAGAGAGATGAAGATGGTAAAGAGCTTGCTGGAGCTACTATGGAACTACGTGATAGTTCTGGAAAAACAATTAGTACTTGGATTTCTGATGGACAAGTTAAAGATTTCTACCTTTACCCGGGTAAATACACATTCGTAGAAACTGCTGCTCCGGATGGATACGAGGTTGCTACAGCTATTACATTCACAGTAAACGAACAAGGTCAAGTTACAGTAAACGGTAAAGCTACTAAGGGAGATGCTCATATTTAA 3’


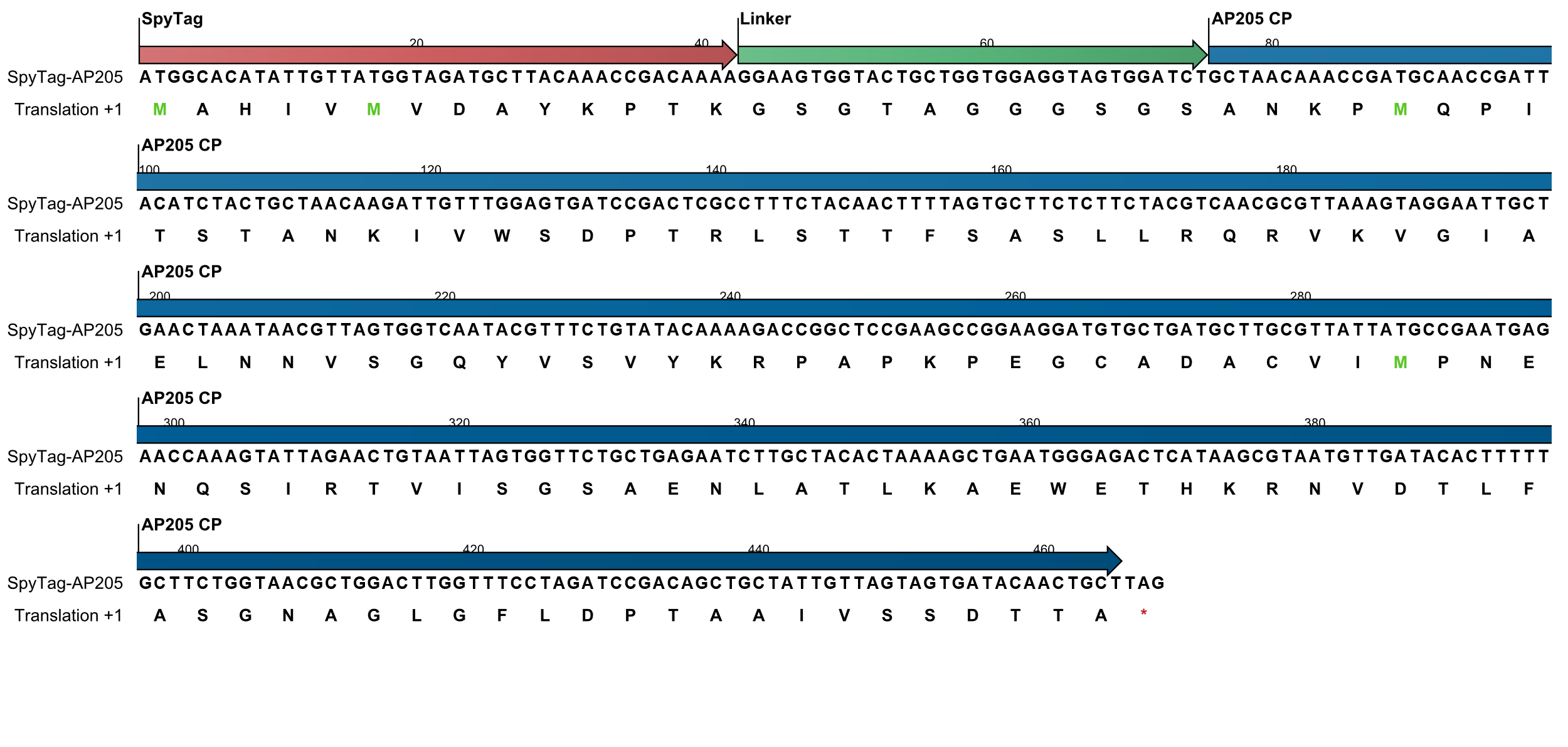


**B)**

5’ATGGCACATATTGTTATGGTAGATGCTTACAAACCGACAAAAGGAAGTGGTACTGCTGGTGGAGGTAGTGGATCTGCTAACAAACCGATGCAACCGATTACATCTACTGCTAACAAGATTGTTTGGAGTGATCCGACTCGCCTTTCTACAACTTTTAGTGCTTCTCTTCTACGTCAACGCGTTAAAGTAGGAATTGCTGAACTAAATAACGTTAGTGGTCAATACGTTTCTGTATACAAAAGACCGGCTCCGAAGCCGGAAGGATGTGCTGATGCTTGCGTTATTATGCCGAATGAGAACCAAAGTATTAGAACTGTAATTAGTGGTTCTGCTGAGAATCTTGCTACACTAAAAGCTGAATGGGAGACTCATAAGCGTAATGTTGATACACTTTTTGCTTCTGGTAACGCTGGACTTGGTTTCCTAGATCCGACAGCTGCTATTGTTAGTAGTGATACAACTGCTTAG 3’

**Figure 1.** DNA and protein sequences of the WNV-EDIII-SC and ST-AP205 constructs used in this study.
