# Supplementary material for: A Plant-Produced Virus-Like Particle Displaying Envelope Protein Domain III Elicits an Immune Response Against West Nile Virus in Mice
Source: Front Plant Sci. 2021 Sep 13;12:738619. doi: 10.3389/fpls.2021.738619 (PMC8475786; doi:10.3389/fpls.2021.738619)
Supplement: Supplementary file 2 [file Data_Sheet_2.DOCX]

Supplementary Material

**Figure 2.** Electron micrograph of plant-produced ST-AP205 VLPs. Scale bar = 50 nm.
