# Supplementary material for: A Plant-Produced Virus-Like Particle Displaying Envelope Protein Domain III Elicits an Immune Response Against West Nile Virus in Mice
Source: Front Plant Sci. 2021 Sep 13;12:738619. doi: 10.3389/fpls.2021.738619 (PMC8475786; doi:10.3389/fpls.2021.738619)
Supplement: Supplementary file 3 [file Data_Sheet_3.DOCX]

Supplementary Material

**Figure 3.** *In vitro* coupling of purified ST-AP205 VLPs and WNV-EDIII-SC. Coupling reactions were performed at molar ratios of 1:1 and 1:2 ST-AP205 (red arrow) to WNV-EDIII-SC (blue arrow). Successful coupling is revealed by a molecular weight shift to 41.5 kDa for AP205:EDIII (black arrow). The shaded triangle in the schematic represents a single coat protein subunit coupled to WNV-EDIII-SC. AP205: *Acinetobacter* bacteriophage AP205 coat protein. EDIII: West Nile virus envelope domain III. ST: SpyTag. SC: SpyCatcher.
