# Supplementary material for: A Plant-Produced Virus-Like Particle Displaying Envelope Protein Domain III Elicits an Immune Response Against West Nile Virus in Mice
Source: Front Plant Sci. 2021 Sep 13;12:738619. doi: 10.3389/fpls.2021.738619 (PMC8475786; doi:10.3389/fpls.2021.738619)
Supplement: Supplementary file 4 [file Data_Sheet_4.DOCX]

Supplementary Material

**Figure 4**. *In vivo* coupling of co-infiltrated ST-AP205 and WNV-EDIII-SC/CRT. Leaves were harvested at 5 dpi and AP205:EDIII VLPs purified by density gradient ultracentrifugation. Successful coupling is revealed by a molecular weight shift to 41.5 kDa for AP205:EDIII (black arrow, monomer – shaded triangle) and ~58 kDa dimer (green arrow). The shaded triangle in the schematic represents a single coat protein subunit coupling to WNV-EDIII-SC. AP205: *Acinetobacter* bacteriophage AP205 coat protein. EDIII: West Nile virus envelope domain III. ST: SpyTag. Red arrows: ST-AP205 protein subunits (16.5 kDa monomer, 33 kDa dimer, 66 kDa tetramer). Black arrow: 41.5 kDa AP205:EDIII coupled complex monomer. Green arrow: ~58 kDa AP205:EDIII coupled complex dimer – AP205 CP dimer with one couple EDIII protein.
