# Supplementary material for: A Plant-Produced Virus-Like Particle Displaying Envelope Protein Domain III Elicits an Immune Response Against West Nile Virus in Mice
Source: Front Plant Sci. 2021 Sep 13;12:738619. doi: 10.3389/fpls.2021.738619 (PMC8475786; doi:10.3389/fpls.2021.738619)
Supplement: Supplementary file 5 [file Data_Sheet_5.DOCX]

Supplementary Material

**Figure 5.** Comparison of AP205:EDIII yield from co-extraction and co-expression. Co-extraction was performed by the homogenisation of leaves infiltrated individually with ST-AP205 and WNV-EDIII-SC/CRT and co-expression was performed by the co-infiltration of cultures harbouring ST-AP205 and WNV-EDIII-SC/CRT constructs. Leaves were harvested at 5 dpi and the AP205:EDIII VLPs purified by density gradient ultracentrifugation. Total EDIII protein for fractions 6 to 8 was calculated by ELISA and 12 ng total EDIII was loaded into each lane. The AP205:EDIII coupled complex is indicated by the black arrow (41.5 kDa, monomer – shaded triangle) and a dimer consisting of two AP205 CP and one EDIII protein is indicated by the green arrow (~58 kDa). The shaded triangle in the schematic represents a single coat protein subunit coupling to WNV-EDIII-SC. AP205: *Acinetobacter* bacteriophage AP205 coat protein. EDIII: West Nile virus envelope domain III. Black arrow: 41.5 kDa AP205:EDIII coupled complex monomer. Green arrow: ~58 kDa AP205:EDIII coupled complex dimer.
