# Supplementary material for: A Plant-Produced Virus-Like Particle Displaying Envelope Protein Domain III Elicits an Immune Response Against West Nile Virus in Mice
Source: Front Plant Sci. 2021 Sep 13;12:738619. doi: 10.3389/fpls.2021.738619 (PMC8475786; doi:10.3389/fpls.2021.738619)
Supplement: Supplementary file 6 [file Data_Sheet_6.DOCX]

Supplementary Material

**Figure 6.** AP205:EDIII protein quantification by densitometric analysis of an anti-WNV-EDIII western blot. *E. coli* produced WNV-EDIII was used as a protein standard (62 kDa). The concentration of purified AP205:EDIII was determined to be 90 µg/mL. The concentration was calculated from the AP205:EDIII monomer (41.5 kDa) and dimer (~58 kDa – AP205 CP dimer with one coupled EDIII protein) indicated by black arrows. pTRAkc: mock antigen purified the same as AP205:EDIII from leaves infiltrated with culture harbouring empty pTRAkc-ERH vector.
