# Supplementary material for: A Plant-Produced Virus-Like Particle Displaying Envelope Protein Domain III Elicits an Immune Response Against West Nile Virus in Mice
Source: Front Plant Sci. 2021 Sep 13;12:738619. doi: 10.3389/fpls.2021.738619 (PMC8475786; doi:10.3389/fpls.2021.738619)
Supplement: Supplementary file 7 [file Data_Sheet_7.DOCX]

Supplementary Material

**Figure 7.** Co-extraction of leaves infiltrated separately with cultures harbouring ST-AP205 and cultures harbouring WNV-EDIII-SC/CRT at different fresh leaf weight ratios to optimise coupling efficiency. Leaves harvested on 5 dpi were co-extracted at FLW ratios of 1:1, 1:2, 1:3 and 1:4 ST-AP205 to WNV-EDIII-SC and AP205:EDIII VLPs purified by density gradient ultracentrifugation. Proteins in purified fractions (F3-5) were detected with anti-WNV-EDIII (top panel) and anti-WNV-ST-AP205 (bottom panel) antiserum. Uncoupled ST-AP205 CP subunits are indicated by red arrows (16.5 kDa monomer, 33 kDa dimer and 66 kDa tetramer). The AP205:EDIII coupled complex is indicated by the black arrows (41.5 kDa, monomer – shaded triangle) and a dimer consisting of two AP205 CP and one EDIII protein is indicated by the green arrows (~58 kDa). The shaded triangle in the schematic represents a single coat protein subunit coupling to WNV-EDIII-SC. AP205: *Acinetobacter* bacteriophage AP205 coat protein. EDIII: West Nile virus envelope domain III. ST: SpyTag.
